# Atmospheric Microbial Transport to Antarctica and Its Ecological Implications: A Systematic Review

**DOI:** 10.1111/1462-2920.70415

**Published:** 2026-09-11

**Authors:** Rodrigo Goldenberg‐Barbosa, Anna Donato, Dafne Anjos, Letícia Eller, Heitor Evangelista, Cesar Amaral

**Affiliations:** ^1^ Center for Molecular Genetics and Astrobiology, Laboratory of Radioecology and Global Changes State University of Rio de Janeiro Rio de Janeiro Brazil; ^2^ Department of Biophysics and Biometrics State University of Rio de Janeiro Rio de Janeiro Brazil; ^3^ Post‐Graduate Program in Ecology and Evolution State University of Rio de Janeiro Rio de Janeiro Brazil; ^4^ Laboratory of Radioecology and Global Changes State University of Rio de Janeiro Rio de Janeiro Brazil

**Keywords:** aerobiology, Antarctica, atmospheric transport, climate change, microbial dispersal, polar ecosystems, systematic review

## Abstract

Atmospheric transport is hypothesized to be the primary vector connecting isolated Antarctic terrestrial habitats to global microbial pools, yet the mechanisms governing this connectivity still have important gaps. We systematically reviewed the present knowledge on how the atmosphere acts not merely as a passive conduit, but potentially as a selective ecological filter and a particular ecosystem. Analysis of 53 studies reveals a three‐pathway framework: (1) long‐range advection via the free troposphere, (2) marine aerosolization from the Southern Ocean, and (3) local resuspension of soils, rock outcrops and cryoconite. Crucially, we identify indirect evidence suggesting that atmospheric transport imposes a strict selection regime dominated by UV irradiation and desiccation. This potential barrier likely ensures that successful colonizers are disproportionately enriched for stress‐tolerance traits, including DNA repair and cold‐active metabolism. Consequently, atmospheric deposition may not just add biomass; it could actively shape Antarctic ecosystems by seeding them with functionally distinct, stress‐adapted pioneers capable of altering local nutrient cycling. We conclude that future research must move beyond simple detection to focus on the viability and metabolic competence of these airborne immigrants to predict their role in a warming Antarctic.

## Graphical abstract

Atmospheric transport to Antarctica operates via long‐range advection, marine aerosolization, and local resuspension, acting as a strict ecological filter. This selective barrier could actively shape local ecosystems by seeding them with functionally distinct, stress‐adapted microbial pioneers capable of surviving extreme conditions.

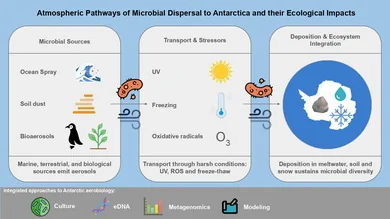

## 1 Introduction

Antarctica's ecosystems are characterized by extreme environmental conditions, geographic isolation, and limited connectivity with other global biomes (Convey et al. 2014). Despite these constraints, microbial communities in Antarctic lakes, surface glaciers, and ice‐free areas exhibit great diversity and complexity (Achberger et al. 2016; Cowan et al. 2014; Karlsson et al. 2020). Understanding how these communities are assembled and maintained has significant implications for predicting ecosystem functionality and responses to climate change.

Atmospheric transport has been recognized as an important mechanism for microbial dispersal (Burrows et al. 2009; Womack et al. 2010). For Antarctica, this process may represent the primary pathway for introducing new genetic material and taxa to isolated terrestrial habitats (Pearce et al. 2009; Bottos et al. 2014; Archer et al. 2019). However, the effectiveness of atmospheric transport depends on multiple factors including the source location, the atmospheric conditions during transport, survival during transit, and successful establishment in recipient ecosystems (Pearce et al. 2016). Advances in molecular techniques and atmospheric sampling methods have enabled more detailed investigations of airborne microbial communities in polar regions (Bottos et al. 2014; Pearce et al. 2016; Roldán et al. 2020; Dieser et al. 2024). These studies have revealed complex patterns of microbial transport that challenge simple models of neutral dispersal and highlight the importance of selection processes during atmospheric transit.

The Southern Ocean represents a unique atmospheric environment that may both facilitate and limit microbial transport to Antarctica (Burrows et al. 2009; Pearce et al. 2009; Archer et al. 2019; Malard et al. 2022). During specific conditions, the circumpolar current system creates distinct boundary layer characteristics that isolate Antarctic air masses derived from surrounding continents while marine‐derived inputs prevail (Burrows et al. 2009; Archer et al. 2019). Better describing these atmospheric dynamics is crucial for identifying sources and interpreting patterns of microbial diversity and endemism in Antarctic terrestrial ecosystems.

Despite growing interest in Antarctic atmospheric microbiology, the field lacks comprehensive synthesis of current knowledge and identification of key research gaps. This systematic review aims to: (1) synthesize current understanding of atmospheric microbial transport mechanisms to Antarctica; (2) evaluate evidence for microbial survival and adaptation during atmospheric transport; (3) assess ecological impacts of atmospheric deposition on Antarctic terrestrial ecosystems; and (4) identify critical knowledge gaps and research priorities for future investigations.

## 2 Methods

We conducted comprehensive searches of two databases: PubMed and Scopus. The search strategy was developed to capture studies investigating atmospheric microbial processes in Antarctic environments. Combined search terms for both databases are detailed in Table S1. We included English language studies from 1991 to 2026 investigating airborne microorganisms in Antarctic environments; research on atmospheric transport of microbes to/from Antarctica; studies on microbial aerosols in the Antarctic atmosphere; research exploring survival mechanisms of atmospheric microbes in polar conditions; studies examining ecological impacts of airborne microorganisms in Antarctic ecosystems; original research articles, systematic reviews, and meta‐analyses.

**FIGURE 1:**
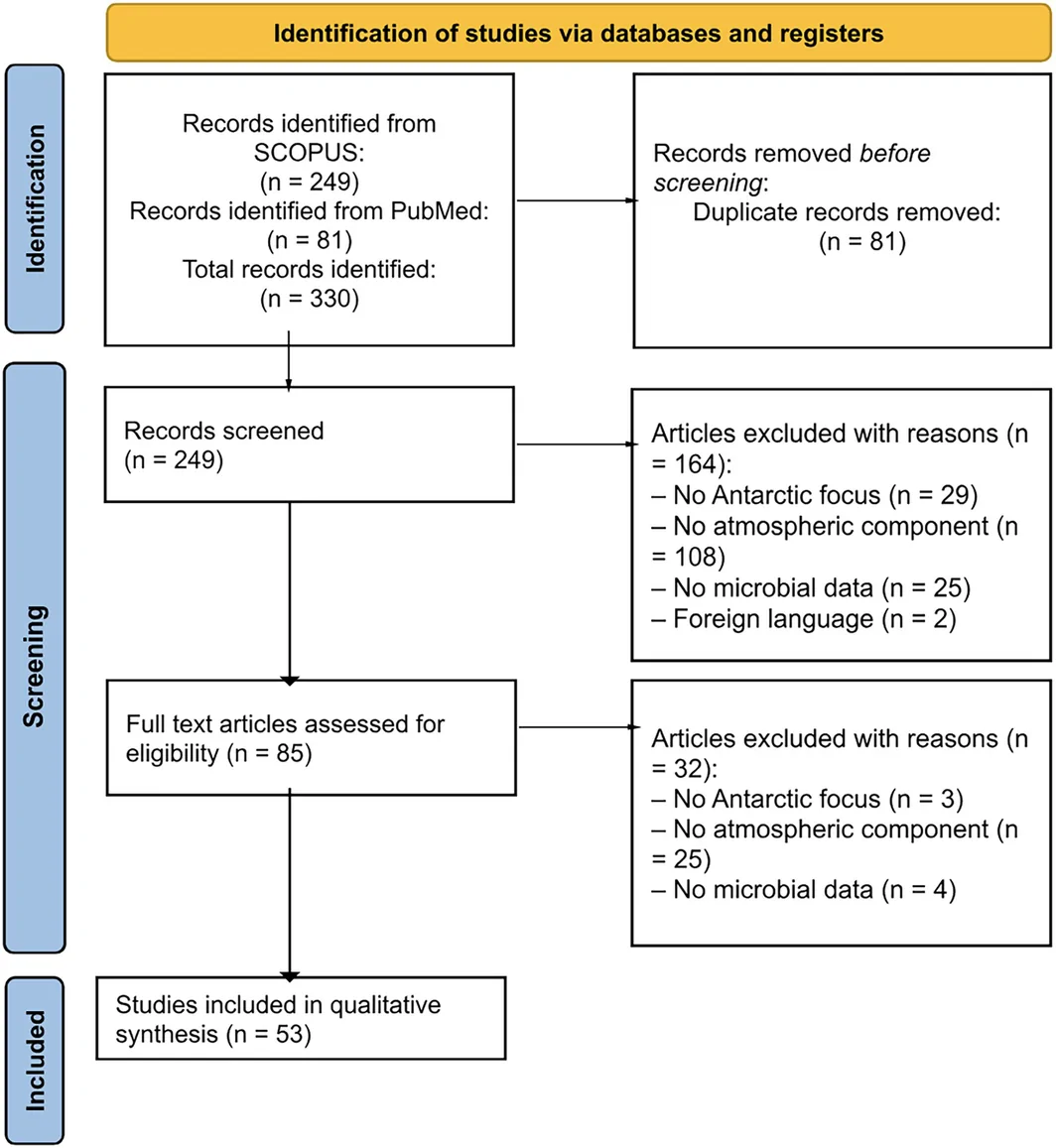
PRISMA 2020 Flow‐diagram showing the systematic review process for Antarctic atmospheric microbiology studies. The diagram illustrates the identification, screening, eligibility assessment, and final inclusion of studies from Scopus and PubMed databases following established systematic review guidelines.

This search yielded 249 articles from Scopus and 81 from PubMed (total = 330). After duplicate removal, 249 unique records remained. Titles and abstracts were screened for relevance, followed by the exclusion of 164 articles (Figure 1). Of the remaining studies, 85 were assessed by full text, with 53 articles included in the qualitative synthesis for this review. Studies were included if they explicitly addressed atmospheric microbial transport, survival, or deposition in Antarctic contexts. Studies focusing exclusively on non‐Antarctic regions or unrelated microbiological processes were excluded. Certain studies that did not meet our inclusion criteria were nonetheless incorporated into our discussion, as they offered valuable contextual information relevant to our analysis.

The 53 included articles were analysed and grouped into four themes: transport mechanisms, survival and adaptation, ecological impacts, and research gaps. Additional relevant Antarctic aerobiology studies were included for context, and some excluded papers are briefly noted for their importance. The review followed the PRISMA 2020 statement (Page et al. 2021). The study selection process (Figure 1) and data extraction details (Table 1; Tables [Link], [Link], [Link], [Link]) ensure transparent reporting and reproducibility.

**TABLE 1:** Representative studies included in this systematic review, illustrating the main atmospheric transport pathways of microorganisms to Antarctica. The table summarizes study location, method, key findings, and evidence for microbial viability.

Study (Author, Year)	Region/site	Sample type and method	Key finding	Pathway	Viability evidence
Dall'Osto et al. 2022	Antarctic Peninsula	Air; DNA‐based + Chemical/Geochemical	Sea‐ice microbiota influence composition of cloud‐relevant aerosols; microbes and organic matter entrained into the atmosphere	Marine aerosolization	No
Malard et al. 2022	Southern Ocean	Air; DNA‐based	Extremely low airborne bacterial biomass; communities structured by meteorology and selective dispersal filtering; Antarctic air distinct but connected to Southern Ocean sources.	Marine aerosolization	No
Junge and Swanson 2008	West Antarctica	Snow/Ice; Culture‐based + Biogeochemical Assay	Polar sea‐ice bacteria display strong ice‐nucleating activity active at high sub‐zero T; likely influence atmospheric ice formation once aerosolized.	Marine aerosolization	Yes
Bera et al. 2012	East Antarctica	Lichen patch scrapings; Microscopy	Non‐native pollen and fungal spores detected in Antarctic lichen patches; authors conclude they were transported from lower latitudes by atmospheric circulation.	Long‐range advection	No
González‐Toril et al. 2009	East Antarctica	Air; Cultured‐based + DNA‐based	Viable pigmented bacteria isolated from Antarctic air; taxa shared with Alpine and Anden snow, showing intercontinental airborne transport	Longe‐range advection	Yes
Dieser et al. 2024	West Antarctica	Air; Cultured‐based + DNA‐based	Demonstrates that airborne bacteria can be cultured from high‐elevation Antarctic glacier aerosols and that these isolates carry genomic traits relevant to survival in cold, oligotrophic environments. Abstract explicitly attributes seeding of these sites to aeolian transport from local or distant ice‐free environments.	Long‐range advection	Yes
Duncan et al. 2010	High Plateau/Interior	Air; Culture‐based + Microscopy	Air inside and outside historic huts contained viable airborne fungal spores; several taxa likely introduced by wind or human presence.	Local resuspension	Yes
Loperena et al. 2012	Antarctic Peninsula	Air + Soil + Snow/Ice; Culture‐based + DNA‐based + Biogeochemical Assay	Psychrotrophic and halotolerant bacteria isolated from particles suspended in air produced cold‐active hydrolyc enzimes (lipase, protease, cellulase). Indicates atmospheric dispersal of viable, metabolically active taxa.	Marine aerosolization/Local resuspension	Yes
Bottos et al. 2014	West Antarctica	Air + Soil; DNA‐based	Found distinct airborne microbial communities above Dry Valley desert soils, showing both local resuspension and long‐range transport signatures. Airborne assemblages partially overlap with local soils, suggesting atmospheric connectivity between sites.	Local resuspension	No
Menes et al. 2021	Antarctic Peninsula	Air; Culture‐based + Biogeochemical Assay	Describes *F. asaccharolytica* as a novel psychrophile isolated from Antarctic air, producing cold‐active hydrolases. Demonstrates that enzyme‐competent, cold‐adapted bacteria are transported and remain viable in Antarctic air masses.	Atmospheric deposition	Yes
Price et al. 2009	High Plateau/Interior	Snow/Ice; Atmospheric Modelling	Direct atmospheric deposition quantified—microbial flux calculated from airborne delivery to Antarctic ice	Atmospheric deposition	No
Câmara et al. 2025	Antarctic Peninsula	Air + Snow/Ice; DNA‐based	Demonstrated shared taxa between air and snow communities, indicating active atmospheric transport and deposition of Antarctic microeukaryotes	Atmospheric deposition	No

## 3 Results

Following screening, 53 studies met inclusion criteria and were included in the qualitative synthesis. Table 1 summarizes 12 representative studies illustrating the main mechanisms studied of airborne microbial transport in Antarctica. These 12 studies were specifically selected to provide a balanced representation of the three primary transport pathways, diverse geographic samplings, and the variety of methodological approaches currently being utilized in the field. The complete dataset is provided in Table S2.

**FIGURE 2:**
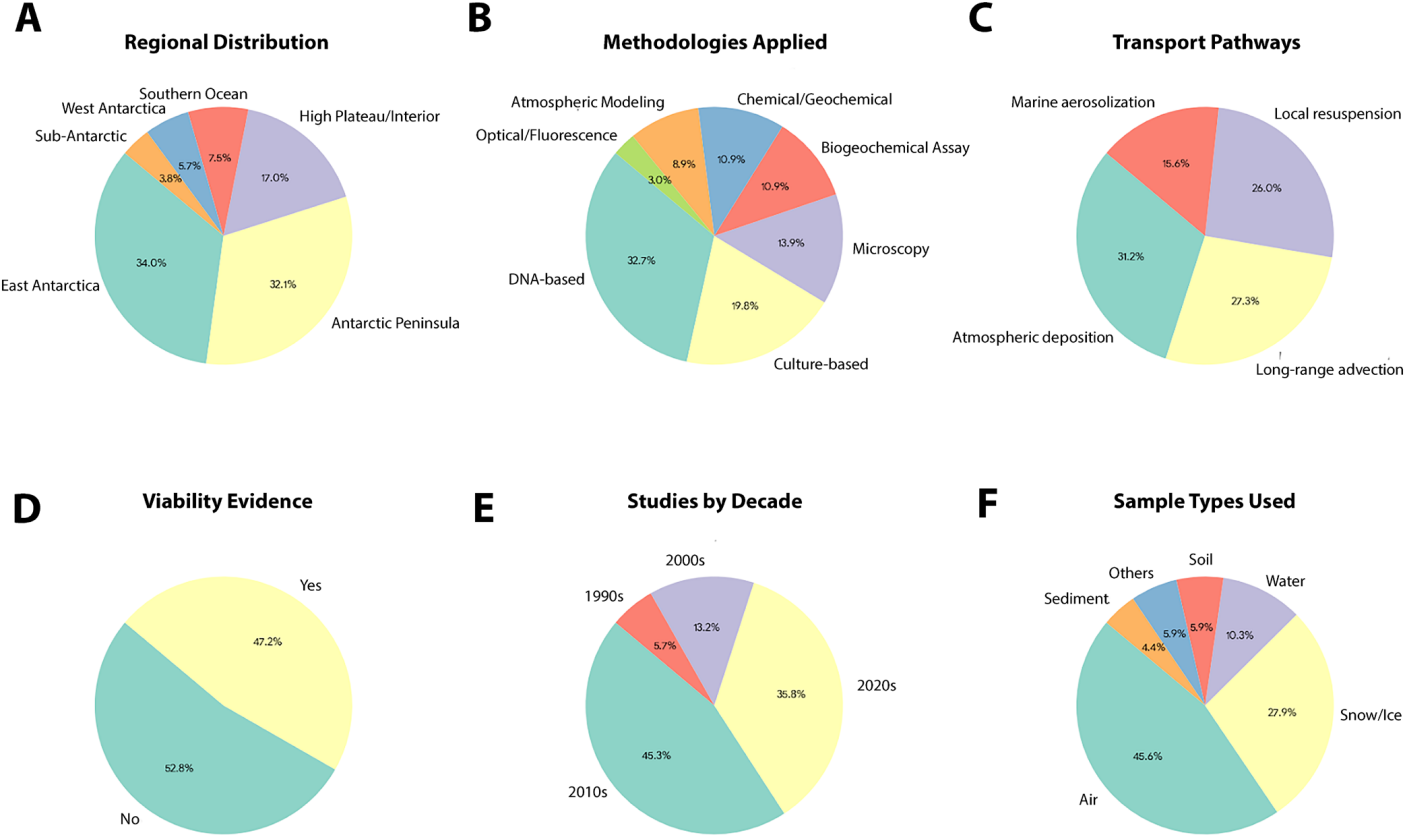
Study characteristics of the 53 research articles included in this systematic review on atmospheric microbial transport to Antarctica. Sector graphics summarize (A) geographic distribution of sampling locations, (B) methodological approaches, (C) pathways studied, (D) viability assessment, (E) temporal distribution by decade, (F) sample types.

Geographically, studies were concentrated on accessible coastal regions, with East Antarctica accounting for ~34% and the Antarctic Peninsula combined with sub‐Antarctic islands comprising ~36% (32% and 4% respectively). Fewer investigations focused on the high plateau interior (~17%) and West Antarctica (~6%), while ~8% of the studies targeted the Southern Ocean (Figure 2A). Regarding methodology, among the total analytical approaches recorded, 32.7% were molecular sequencing (DNA‐based), 19.8% were culture‐based techniques, 13.9% were microscopy, 10.9% were biochemical assays, 10.9% were chemical/geochemical analyses, 8.9% were atmospheric modelling, and 3.0% utilized optical/fluorescence instrumentation (Figure 2B). Of the transport pathways investigated, 31.2% focused on atmospheric deposition, 27.3% on long‐range advection, 26.0% on local resuspension, and 15.6% on marine aerosolization (Figure 2C). Slightly more than half of the studies (52.8%) did not provide direct evidence of microbial viability (Figure 2D). The included studies spanned from 1991 to 2026, with the vast majority conducted in the last two decades (Figure 2E). Finally, regarding sample types, air samples were the most common (45.6%), followed by snow and ice (27.9%), water (10.3%), soil (5.9%), others (rain, dust, lichen) (5.9%), and sediment (4.4%) (Figure 2F).

Taken together, our synthesis reveals pronounced spatial, methodological, and conceptual imbalances in the study of microbial transport to Antarctica. There is a strong emphasis on accessible coastal regions, atmospheric sampling, and molecular detection approaches, while large areas of the continental interior, benthic and terrestrial sinks, and viability‐focused assessments remain comparatively understudied. These patterns suggest that current knowledge may be biased toward detection rather than ecological function and persistence, limiting our capacity to evaluate the true environmental significance and implications of microbial dispersal to and within Antarctic ecosystems.

## 4 Discussion

### 4.1 Atmospheric Transport Mechanisms

Antarctica's aerobiological connectivity emerges from the interplay of hemispheric circulation, marine aerosolization, and local resuspension (Rosa et al. 2020; Zeppenfeld et al. 2021; Cataldo et al. 2023). These three origins of microbial dispersion can be directly linked to transportation within different atmospheric layers: long‐range advection is primarily associated with above‐cloud transport via the free troposphere (Kobayashi 2022, 2025), marine aerosolization is coupled to in‐cloud and marine boundary layer processes (Sanchez et al. 2021; Dall'Osto et al. 2022), and local resuspension operates below‐cloud in the low‐altitude boundary layer (Galbán et al. 2025; Rosa et al. 2020). Air‐mass analyses along meridional transects and station‐based time series demonstrate that long‐range advection serves as a primary vector, where incursions from other continents coincide with pulses of airborne microbial diversity (da Matta Agostini et al. 2017; Cataldo et al. 2023). Pollen grains retrieved from lichen patches further corroborate these long‐distance biotic connections, serving as durable tracers of continental wind pathways (Bera et al. 2012). This pattern is established in foundational coastal‐to‐interior surveys (Parro et al. 2025) and supported by observations spanning multiple austral summers that resolve repeated advection corridors linking Patagonia and the Antarctic Peninsula, for instance (Cataldo et al. 2023). Complementary evidence from cryosphere archives shows that these atmospheric inputs are preserved as stratified deposition signals; the snowpack acts as a reservoir that selects cold‐adapted taxa over time (Parro et al. 2025). Coastal snow layers occasionally retain cyanobacterial lineages traceable to low‐latitude sources, indicating efficient poleward displacement during favourable synoptic windows (Parro et al. 2025).

In contrast to the extensive connectivity observed with South America, the atmospheric pathway from Australasia appears significantly more constrained, though this may partially reflect that it is currently less explored rather than strictly less frequent. While the migration of cyclonic systems and the Andes mountain range disrupt the Southern Hemisphere westerlies to facilitate rapid, meridional transport corridors across the relatively narrow Drake Passage (Garreaud 2009; Hoskins and Hodges 2005), the Australian pathway lacks a direct transport to the higher latitudes. Historically, this apparent constraint has been attributed to the greater distance from the land to the Antarctic continent. However, it has been demonstrated that air trajectories arriving to Antarctica do not follow direct routes and can arrive from very distant land masses (Stohl and Sodemann 2010). During this prolonged journey, the already limited terrestrial aerosols are subjected to intense precipitation scavenging, or “washout”, by persistent marine storm systems (Sanchez et al. 2021). Comprehensive sampling of the Southern Ocean boundary layer south of Tasmania reveals that, even when air mass trajectories originate from the Australian continent, the bioaerosol load remains overwhelmingly marine (Uetake et al. 2020). Consequently, during the austral summer, the marine boundary layer serves less as a bridge and more as a barrier to passive microbial influx from the Australasian sector (Uetake et al. 2020).

This barrier effect may be strictly limited to the lower boundary layer. Unmanned aerial vehicle (UAV) based and tethered balloon sampling over East Antarctica (S17 Base and Syowa Station) revealed distinct microbial stratification, where upper‐air masses (approx. 1000 m) contained specific taxa (e.g., *Arcobacter*, *Finegoldia*) absent from the local ice surface (Kobayashi 2022, 2025). Backward trajectory analyses linked these upper‐air assemblages to long‐range transport via the free troposphere (up to 4500 m altitude), suggesting that while the boundary layer filters inputs, high‐altitude advection remains a permeable corridor for continental taxa (Kobayashi 2025).

An historical and conceptual arc runs through station and traverse work documenting that the circumpolar westerlies and polar front act as semi‐permeable, rather than absolute barriers. Deep ice core analysis provided some of the earliest evidence for this permeability, linking discrete microbial deposition layers to atmospheric transport events that vary with climate history (Segawa et al. 2010; Parro et al. 2025). Complementary research on ice sheet fluxes further established that microorganisms and organic aerosols are deposited onto the ice surface at rates consistent with atmospheric delivery, effectively archiving these transit events (Price et al. 2009). Foundational aerobiological surveys anticipated these findings by explicitly connecting airborne propagules to the colonization of exposed fellfield soils, demonstrating the wind's role in dispersing biological material across the continent (Wynn‐Williams 1991). More recent work has refined this understanding by characterizing the specific composition of these inputs; for instance, studies of snow and aerosol samples have successfully cultured diverse bacterial communities that confirm the viability of microbes during dispersal (González‐Toril et al. 2009). Furthermore, targeted monitoring of airborne fungi has distinguished between local inputs and long‐range transport, validating the arrival of biological material from distant sources (Duncan et al. 2010). This biological inventory has recently been expanded to include non‐fungal eukaryotes; DNA metabarcoding confirms that diverse assemblages of microalgae and protists are also present in Antarctic air masses, travelling alongside bacteria and fungi (Câmara et al. 2025). Finally, investigations into the snow surface microbiome continue to support these mechanisms by linking bacterial diversity directly to atmospheric transport vectors (Michaud et al. 2014; Cataldo et al. 2023).

Marine aerosolization provides a second, persistent pathway. Sea‐spray entrainment and bubble‐bursting at the ocean surface inject primary biological aerosol particles and organic‐rich film material into the marine boundary layer, which then feeds the Antarctic free troposphere (Dall'Osto et al. 2017, 2019; Zeppenfeld et al. 2021). As bacterial communities are transferred directly from the sea‐surface microlayer (SML) into the atmosphere, the specialized composition of the SML starts acting as a key factor for regional microbial dispersion (Martinez‐Varela et al. 2020). In the South Shetland Islands, the SML naturally concentrates surface‐active organic matter, becoming highly enriched in bacteria capable of degrading anthropogenic organic pollutants (e.g., *Pseudoalteromonas*, *Colwellia*), correlating with concentrations of perfluoroalkyl substances (PFAS). By concentrating these specific taxa at the very interface of the ocean and atmosphere, the SML primes them for aerosolization (Martinez‐Varela et al. 2020). Taxonomic surveys at Byers Peninsula support this marine‐to‐atmosphere bridge, detecting families such as *Colwelliaceae* and *Granulosicoccaceae*, as well as genera like *Polaribacter*, in air samples. These taxa are typical of Antarctic coastal waters, confirming that marine aerosols actively seed the coastal aerobiome with viable marine lineages (Galbán et al. 2025). Conversely, wider surveys of the Southern Ocean boundary layer reveal a remarkably pristine bacterial signal dominated by marine taxa. This suggests that, in summer, when a seasonal minimum in north‐to‐south air flow combined with the isolating barrier of westerly circumpolar winds prevents continental air masses from reaching the Southern Ocean, continental bioaerosols are largely filtered out before reaching the lower atmosphere (Uetake et al. 2020).

Specific chemical tracers reveal that this process is highly selective; carbohydrates and polysaccharides are enriched in submicron particles (Zeppenfeld et al. 2021), while sea ice microbiota, including bacteria, protists, and viruses, actively modulate the production of sea spray aerosol (Dall'Osto et al. 2022). Multi‐platform observations document amines and organic nitrogen species co‐occurring with these biological particles during high sea‐salt episodes, pointing to oceanic microlayers as recurrent sources of airborne inocula (Dall'Osto et al. 2017, 2019, 2022; Zeppenfeld et al. 2021). Broader assessments around the Southern Ocean confirm that air masses transiting high‐productivity waters deliver only sparse microbial biomass, yet the assemblages are consistently structured rather than random (Malard et al. 2022).

Local resuspension within Antarctica constitutes a third mechanism, redistributing microorganisms across ice‐free landscapes and coastal margins (Rosa et al. 2020). High‐wind events mobilize soil, lithic, and cryoconite particles, reintroducing previously deposited cells into the air column and promoting short‐range exchange among neighbouring habitats (Rosa et al. 2020). Observations of lithic‐associated and station‐proximal microbiota in air imply that near‐surface sources seed the boundary layer, producing measurable atmospheric signatures of adjacent ground communities (Sterflinger et al. 2012). Recent quantitative comparisons at Byers Peninsula have solidified the dominance of this pathway, revealing that 79.4% of airborne Amplicon Sequences Variants (ASVs) were shared with local soils (Galbán et al. 2025). This extensive overlap is driven heavily by spore‐forming bacteria, such as *Paenibacillaceae*, alongside cold and radiation‐resistant taxa like *Gemmatimonadaceae* and *Moraxellaceae* (specifically the genus *Psychrobacter*). The successful aerosolization of these resilient edaphic residents identifies local soils as the net primary contributor to the low‐altitude airborne community in coastal ice‐free areas, potentially overshadowing long‐range inputs during periods of low wind stress (Galbán et al. 2025).

**FIGURE 3:**
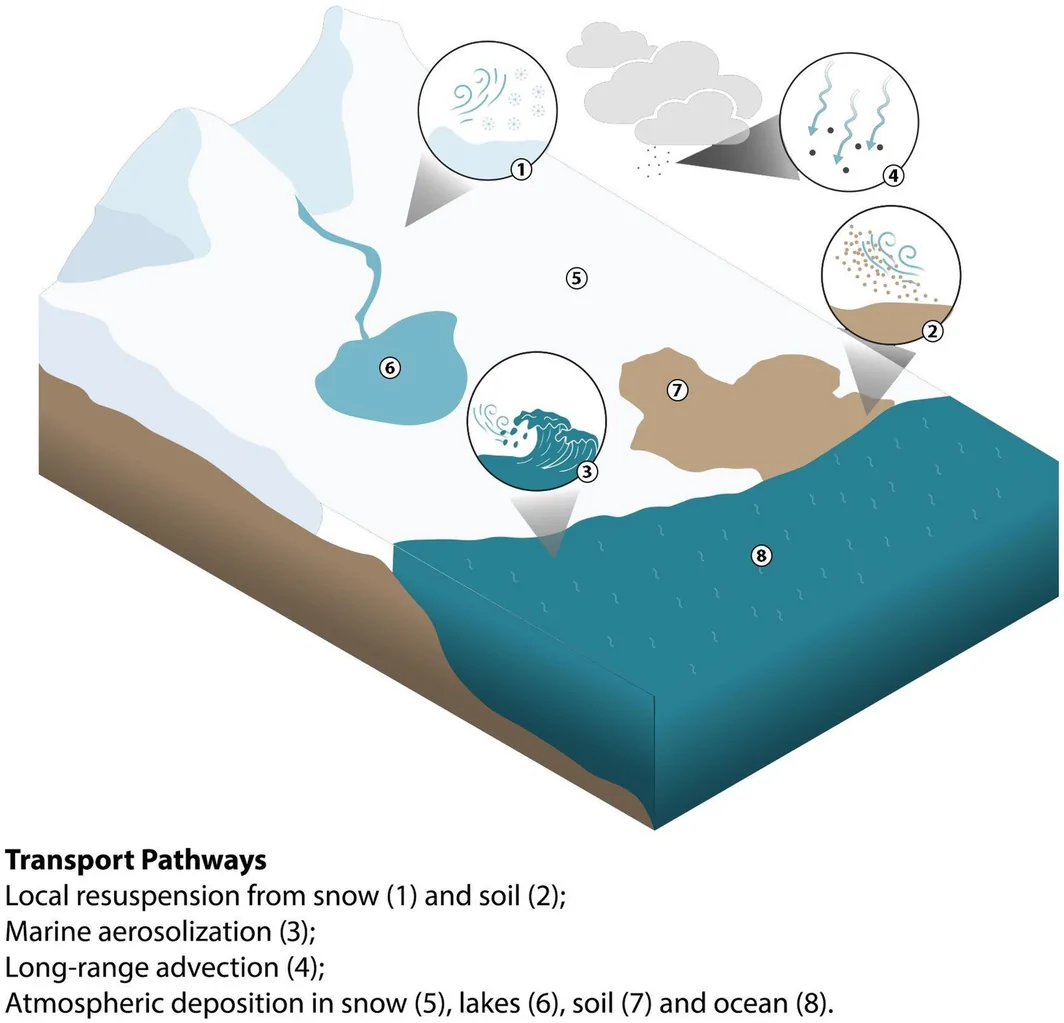
A graphical depiction of microbial transport mechanisms in Antarctica. The diagram illustrates the three principal pathways governing the input and redistribution of microorganisms: Long‐Range Advection (from continental sources, e.g., South America, via the free troposphere); Marine Aerosolization (continuous input from sea‐spray and the sea‐surface microlayer of the Southern Ocean); and Local Resuspension (recycling of cells from Antarctic soil, cryoconite, and lithic substrates).

**FIGURE 4:**
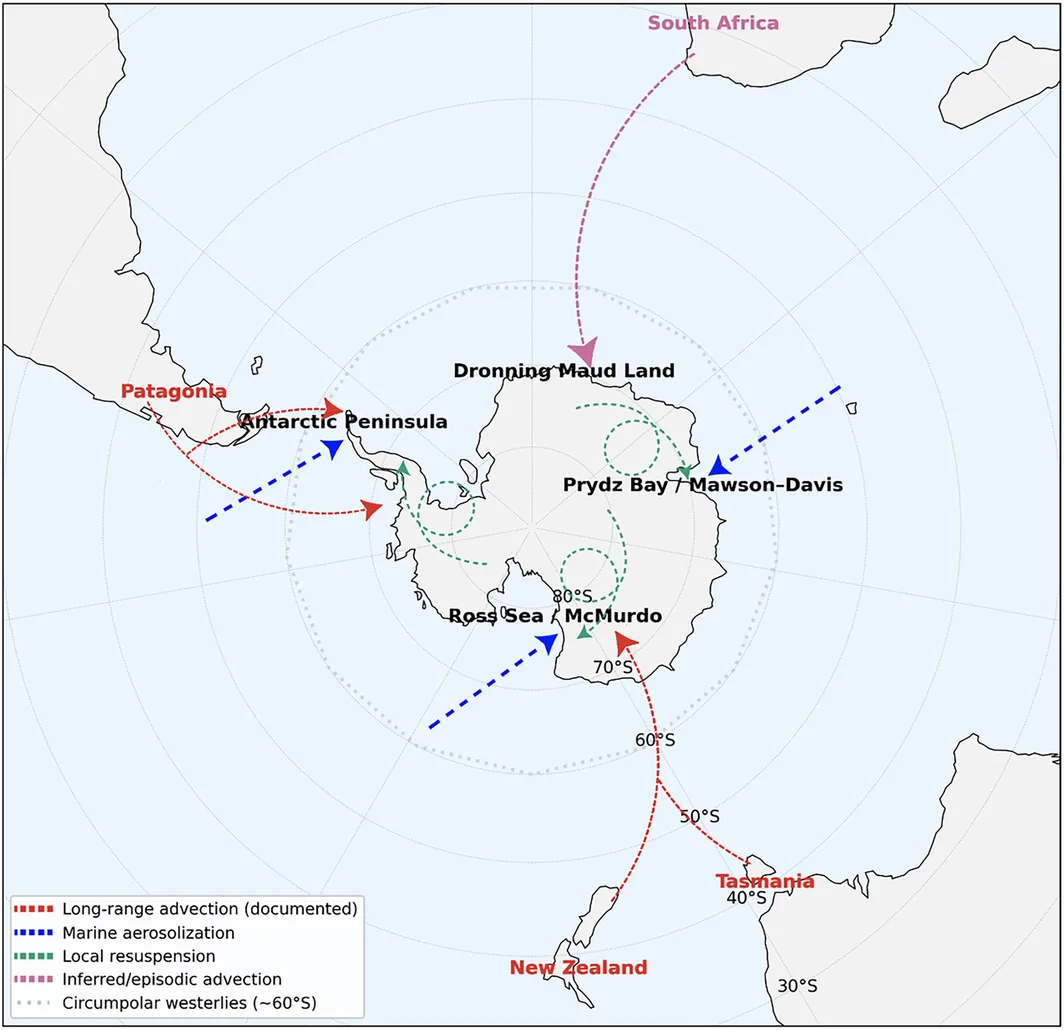
Map of Antarctica showing major atmospheric microbial transport pathways. Schematic map illustrating the primary pathways delivering airborne microorganisms to Antarctica. Long‐range advection (red) represents well documented episodic air‐mass incursions from South America (da Matta Agostini et al. 2017; Cataldo et al. 2023) and Australasia (Uetake et al. 2020) that cross the circumpolar westerlies. Inferred episodic air mass incursion (magenta) from South Africa (Parro et al. 2025). Marine aerosolization (blue) indicates continuous sea‐spray injection and ocean–atmosphere coupling around the Southern Ocean (Dall'Osto et al. 2017, 2019, 2022; Zeppenfeld et al. 2021; Malard et al. 2022; Boyer et al. 2025). Local resuspension (green) depicts recycling of microorganisms from Antarctic soils, cryoconite, and coastal margins under high wind stress (Sterflinger et al. 2012; Hodson et al. 2017; Rosa et al. 2020). The map was generated using the Cartopy library (v0.22) with the South Polar Stereographic projection.

In the Untersee Oasis, a potential recycling loop has been proposed: ‘lift‐off’ microbial mats occasionally detach from the lake floor and float to the surface, and these escaped mats may be transported by wind toward the adjacent Anuchin Glacier, where they could contribute to seeding cryoconite habitats that, in turn, supply microbial inputs back to the lake (Weisleitner et al. 2019). Although wind‐driven, this localized dispersal of macroscopic mat fragments occurs primarily near the ground and over short distances, functioning distinctly from long‐range atmospheric transport (Weisleitner et al. 2019; Rosa et al. 2020). The broader ecological role of such particle‐attached microorganisms remains an important area for future investigation to better understand near‐surface dispersal dynamics (Hodson et al. 2017). Process‐oriented work on supraglacial and proglacial surfaces supports this interpretation by documenting the ready entrainment of biologically active particulates from cryoconite and fine sediments under wind stress, thereby sustaining a local recycling loop that operates alongside long‐range inputs (Hodson et al. 2017). These local inputs are corroborated by molecular inventories from coastal and interior stations, which document specific bacterial and fungal taxa, such as *Hymenobacter* and *Sphingomonas*, in both aerosols and snowpack layers (Busse et al. 2003; Antony et al. 2012; Loperena et al. 2012; Lopatina et al. 2013; Roldán et al. 2020, 2021; Menes et al. 2021). However, caution is required when interpreting station‐based data; aerobiological surveys at Halley Station revealed that up to one‐third of detected sequences were closely related to human‐associated microorganisms, suggesting that research facilities may contribute a localized anthropogenic signal that can complicate the interpretation of natural microbial inputs in ultra‐low biomass environments (Pearce et al. 2010). Major atmospheric transport pathways delivering microorganisms to and within Antarctica are summarized in Figures 3 and 4.

Several studies contextualize these biological observations within the broader physics and chemistry of atmospheric transport. Chemical transport modelling and back‐trajectory reconstructions clarify source regions, residence times, and vertical mixing, helping to parse marine versus continental contributions during synoptic passages (Kobayashi et al. 2016; Kobayashi 2022, 2025; Moallemi et al. 2021). Aerosol chemistry campaigns and long‐term records reveal that organic carbon and secondary organic matter co‐vary with periods of enhanced advection, conditions that also favour biological particle transport (Barbaro et al. 2015; Decesari et al. 2020). Additionally, real‐time measurements have linked these transport events to pulses in fluorescent biological particles (Crawford et al. 2017). These chemical matrices may indirectly modulate microbial persistence in air; for instance, airborne organic pollutants have been shown to impact the viability of natural microbial communities, thereby shaping the survival filter applied during transit (Echeveste et al. 2024). Genomic characterization of a limited panel of seven airborne isolates provides compelling mechanistic evidence for their unique stress adaptations, including specific genes for cold and oxidative stress resistance (Dieser et al. 2024). Extrapolating the adaptive capacity of a few cultivated strains to the complete airborne community remains a challenge that requires broader metagenomic validation.

### 4.2 Survival and Adaptation During Atmospheric Transport

Microbial persistence in the Antarctic atmosphere is governed by a multitude of stressors, including intense ultraviolet irradiance, desiccation, subzero temperatures, freeze–thaw cycling, and oxidant exposure. Together, these factors impose a powerful selection filter that shapes airborne communities (Antony et al. 2012; Bottos et al. 2014; Malard et al. 2022). Convergent evidence from molecular surveys and functional inference demonstrates that Antarctic airborne assemblages are not stochastic draws from upwind sources but are selectively enriched for resilience traits (Bottos et al. 2014; Malard et al. 2022). Genomic analyses highlight the overrepresentation of specific genes for cold shock, osmotic stress, and oxidative‐stress defences, consistent with survival under high UV and energy limitation during transport (Menes et al. 2021; Dieser et al. 2024). Similarly, metabarcoding studies along the Antarctic coast confirm that airborne consortia represent ecologically filtered subsets of regional microbial pools (Bottos et al. 2014; Malard et al. 2022).

Survival strategies at the cellular scale involve coordinated remodelling of lipid composition to maintain membrane fluidity, as well as the accumulation of compatible solutes to stabilize proteins and DNA (Antony et al. 2012; Loperena et al. 2012; Dieser et al. 2024). Specific compatible solutes, such as sucrose and trehalose, have been identified in viable Antarctic cyanobacteria, likely contributing to their freeze–thaw tolerance (Parro et al. 2025). Structural adaptations also play a critical role; spore formation and small, desiccation‐resistant cell morphologies reduce water loss and increase aerodynamic persistence, while extracellular polysaccharides provide physical shielding and hygroscopic microenvironments (Busse et al. 2003; Bottos et al. 2014; Antony et al. 2012; Lopatina et al. 2013). Consistent with this, the airborne ‘core community’ at Byers Peninsula was dominated by *Paenibacillaceae* (approx. 20% of reads), a family of known spore‐formers, alongside cold‐resistant *Moraxellaceae* and *Gemmatimonadaceae* (Galbán et al. 2025). Parallel evidence supports a marine conduit for robust phenotypes: halophilic Archaea with buoyant gas vesicles and bacteria with hydrophobic cell envelopes, traits advantageous for sea‐spray entrainment and aerosol residence, have been described in strain reports and environmental surveys (DasSarma et al. 2017; Gesheva et al. 2010). Similarly, cryoconite holes on the Anuchin Glacier have been shown to harbour distinct Archaeal populations (*Halobacteria*, *Thermoplasmata*), broadening the known taxonomic range of supraglacial survivors beyond bacteria (Weisleitner et al. 2020).

To accurately assess viability, it is important to clearly discriminate between air‐collected microorganisms in transit and post‐deposition snow isolates, as they represent distinct substrates with different survival dynamics (Rosa et al. 2020). Once airborne cells are deposited into the cryosphere, they transition into a secondary environmental matrix (Michaud et al. 2014; Hodson et al. 2017). While data from snow, ice, and aquatic matrices do not measure in‐flight survival directly, their relationship to the airborne community is critical: viability within these receiving substrates dictates whether aerially transported microbes can successfully resuscitate and colonize the Antarctic ecosystem after deposition (Hodson et al. 2017; Dieser et al. 2024).

Across both of these matrices, solar UV remains the dominant mortality factor. Exposure experiments and field studies demonstrate rapid losses of DNA integrity under austral summer irradiance (Zepp et al. 2003). Field experiments with sewage‐derived aerosols at Rothera Station quantified this risk, showing that moderate solar UV exposure reduced the viability of airborne coliforms by 99.9% within a single hour of deposition, indicating that environmental stresses severely limit persistence following deposition of non‐adapted taxa (Hughes 2003). However, extrapolating these mortality rates from non‐adapted coliforms to natural airborne communities is likely an unjust comparison, as endemic Antarctic taxa possess highly specialized adaptations to mitigate solar radiation (Sterflinger et al. 2012; Antony et al. 2012; Dieser et al. 2024).

Traits recurrently reported across polar lineages include carotenoid and dark pigments, which are well documented in Antarctic isolates and cryospheric microbiota (Sterflinger et al. 2012; Antony et al. 2012; Parro et al. 2025). Field modelling further suggests that ambient UV radiation impacts microbial community structure and increases phytoplankton mortality in Antarctic waters (Nuñez et al. 2006). Despite these pressures, direct demonstrations of viability in polar air and snow underscore that a fraction of transported cells remain metabolically competent. Culture‐positive recoveries consistently include enzyme‐producing heterotrophs and cyanobacteria that retain activity at low temperatures (Loperena et al. 2012; Menes et al. 2021; Antony et al. 2012; Parro et al. 2025). This builds on early Antarctic microbiology work documenting dormancy‐resuscitation cycles (Wynn‐Williams 1991; McKay and Friedmann 1985; Junge and Swanson 2008) and is supported by contemporary observations of rapid metabolic resumption in transient liquid water environments (Hodson et al. 2017; Michaud et al. 2014; Parro et al. 2025).

A persistent methodological challenge is discriminating viable cells from relic DNA in these ultra‐low‐biomass samples. Amplicon surveys in Antarctic air are intrinsically susceptible to trace contamination and cannot, by themselves, resolve physiological state. Calls to integrate cultivation, viability dyes, and single‐cell approaches recur throughout the aerobiology literature, with the aim of quantifying the fraction of metabolically active versus inert genetic material (Bottos et al. 2014; Lopatina et al. 2013; Santibáñez et al. 2018). This distinction is particularly important for cyanobacterial detections in snow layers, where sequence presence may exceed the fraction of cells capable of photosynthetic recovery (Parro et al. 2025).

**FIGURE 5:**
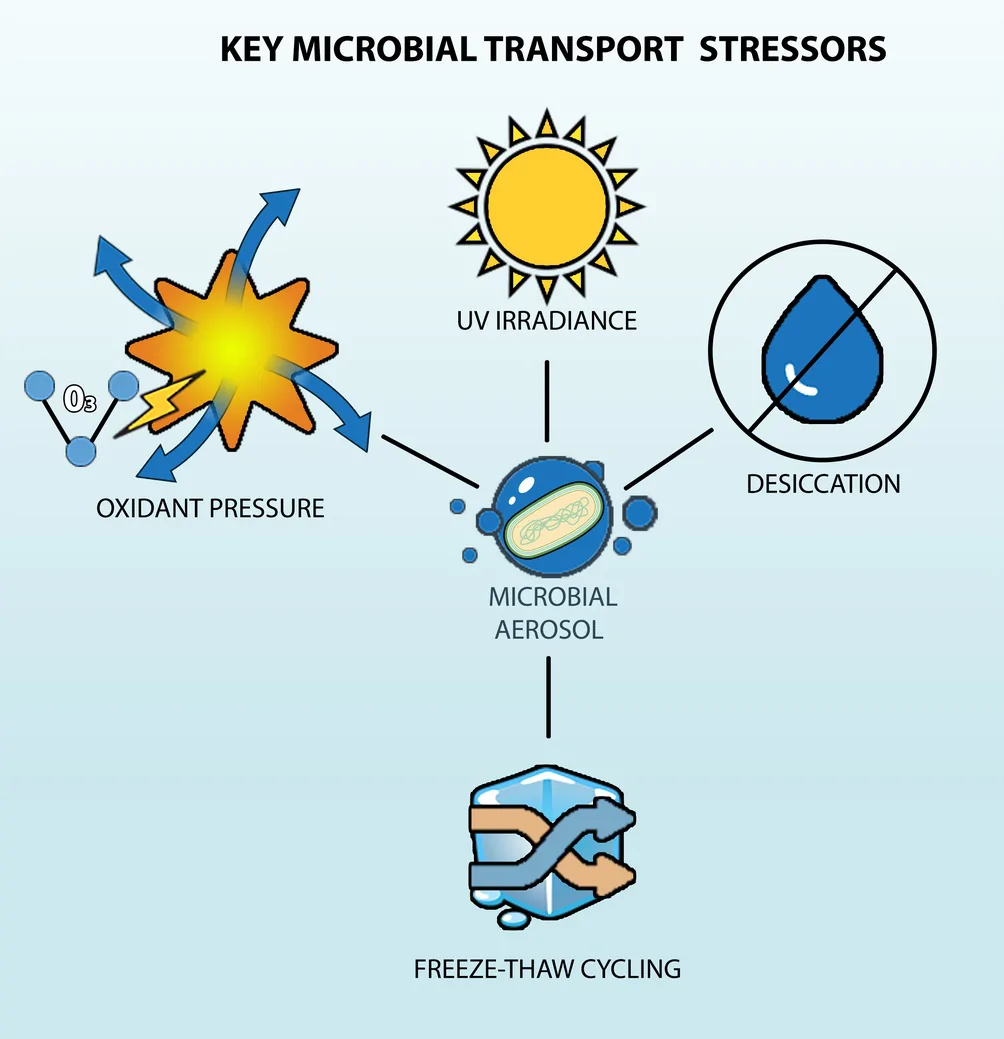
Microbial stressors in the Antarctic atmosphere. Icons represent key selective pressures shaping airborne microbial survival, including UV radiation, oxidative radicals, desiccation, and freeze–thaw cycling.

Finally, survival must be interpreted within its chemical and physical scaffold. Aerosol composition, including amines, organic carbon, and secondary organics, modulates hygroscopicity and atmospheric residence, thereby influencing microbial stress exposures during flight (Barbaro et al. 2015; Crawford et al. 2017; Decesari et al. 2020). Transport dynamics and particle microphysics further determine residence time and UV dose, acting as a selective filter (Kobayashi et al. 2016; Kobayashi 2022). In combination, these factors (Figure 5) explain why the organisms that arrive in Antarctica are disproportionately stress‐adapted and enzyme‐competent (Dieser et al. 2024; Menes et al. 2021), providing a mechanistic bridge between atmospheric selection and ecological function upon deposition.

### 4.3 Ecological Impacts of Atmospheric Deposition

Deposition of microorganisms into Antarctic soils, snow, and freshwater systems represents a critical, albeit low‐biomass, subsidy to local ecosystems. Studies confirm that airborne taxa are actively incorporated into soil assemblages, replenishing microbial diversity in environments otherwise constrained by dispersal barriers (Bottos et al. 2014; Malard et al. 2022). Once established, these inputs functionally alter their new habitats; bacteria producing cold‐active hydrolases, such as lipases, proteases, and amylases, introduce catalytic capabilities that enhance organic matter turnover in oligotrophic cryospheric systems (Loperena et al. 2012; Menes et al. 2021; Parro et al. 2025). Furthermore, metagenomic evidence suggests that some deposited taxa carry the genomic capacity for atmospheric trace‐gas scavenging (H_2_ and CO), providing a critical alternative energy source that supports primary production in carbon‐poor Antarctic soils (Ji et al. 2017).

Beyond direct metabolic contributions, deposition underpins broader ecological networks. Airborne cyanobacteria and melanized fungal spores provide the necessary photobionts and decomposers for the colonization of exposed lithic substrates (Duncan et al. 2010; Sterflinger et al. 2012). Similarly, the deposition of airborne eukaryotic algae and heterotrophic protists may contribute biological inputs relevant to the potential development of more complex microbial communities in snow and ice environments (Câmara et al. 2025). Once established, these communities may engage in complex chemical interactions; for instance, Antarctic marine isolates have been shown to produce volatile organic compounds that actively suppress competitors, suggesting that deposition also introduces chemically mediated competitive traits into local assemblages (Sannino et al. 2017). Furthermore, the delivery of viral particles via sea spray aerosols introduces top‐down controls on these communities, driving dynamics through phage infection and horizontal gene transfer (Dall'Osto et al. 2022). Recent metagenomic work extends this dispersal range significantly, identifying host‐associated prophages of *Ralstonia* and *Janthinobacterium* that are shared between snow on the East Antarctic Plateau and seawater from the Western Antarctic Peninsula, a distance of over 5000 km, implying a pan‐continental dispersal network for phage‐host pairs (Rahlff et al. 2022).

These biological inputs are intimately coupled with nutrient‐bearing aerosols that catalyse ecosystem productivity. While organic carbon in Antarctic snow is primarily associated with marine aerosol input, local microbial biomass contributes to this pool, particularly at inland sites where sea‐salt inputs diminish; notably, crustal dust contributions remain marginal (Antony et al. 2012). Likewise, ammonia emissions from penguin colonies, for example, do not merely influence atmospheric chemistry but actively fertilize nitrogen‐limited terrestrial and snowpack catchments (Boyer et al. 2025). Similarly, episodic volcanic ash and dust events act as abiotic fertilizers, enriching soils with bioavailable trace metals (Hodson et al. 2017). Finally, atmospheric deposition exerts physical feedback on the Antarctic cryosphere itself. Deposited microorganisms and organic detritus darken the snowpack, accelerating melt through reduced albedo (Hodson et al. 2017), while biological aerosols may actively trigger the precipitation that delivers them by serving as efficient ice nucleators (Christner et al. 2008). Over longer timescales, this deposition creates a stratified archive of viable inocula that, upon release during melt periods, seeds microbial mats and productivity hotspots downstream (Michaud et al. 2014; Segawa et al. 2010; Parro et al. 2025). Genera such as *Polaromonas* and *Pseudomonas*, which have been detected in polar air (Weisleitner et al. 2019; Martinez‐Varela et al. 2020), were found to dominate the bacterioplankton of shallow Antarctic lakes, confirming the efficient colonization of aquatic sinks by these airborne dispersers (Michaud et al. 2012). This aerial seeding is particularly pronounced in maritime Antarctic lakes, where environmental gradients drive community assembly, but airborne inputs serve as a continuous source of diversity that could buffer local extinctions (Picazo et al. 2019). Quantitative source‐tracking suggests that supraglacial cryoconite hole communities represent a substantial potential source of benthic microbial mat taxa in Lake Untersee, accounting for up to ~36% of the assemblage (Weisleitner et al. 2019). Together, these findings demonstrate that sporadic atmospheric deposition exerts a disproportionate influence on Antarctic community composition, metabolic potential, and ecological trajectories.

### 4.4 Knowledge Gaps and Research Priorities

The systematic literature highlights several cross‐cutting uncertainties. The most present is the ambiguity around microbial viability: many studies detect genetic material in Antarctic air but cannot discriminate living from relic cells. To address this, recent work has successfully integrated flow cytometry with viability dyes (Santibáñez et al. 2018) and compared rDNA versus rRNA libraries (Lopatina et al. 2013) to distinguish metabolically active fractions from inert transport. However, widespread implementation of these methods remains rare, constraining broader estimates of the ecological significance of deposition.

A second limitation is taxonomic scope. Most Antarctic aerobiology emphasizes bacteria, whereas fungi and viruses, though repeatedly detected, remain underexplored. Fungal spores are consistently found in air and lithic niches (Sterflinger et al. 2012; Duncan et al. 2010), while viral particles have been identified as active modulators of sea spray aerosol properties (Dall'Osto et al. 2022). Recent DNA metabarcoding of air and snow on Livingston Island has begun to address this imbalance by revealing a diverse, non‐fungal eukaryotic assemblage, including algae and protists, highlighting the complexity of airborne dispersal beyond the bacterial domain (Câmara et al. 2025). These eukaryotic spores and viral propagules represent both dispersal agents and community modulators, and their neglect limits understanding of the full ecological reach of deposition. Even where viral signatures are detected over vast spatial scales, metagenomic evidence alone cannot confirm whether these represent viable, ecologically active phage‐host pairs, underscoring that apparent genetic connectivity must be interpreted cautiously (Rahlff et al. 2022; Smith et al. 2013).

A critical spatial blind spot remains the vertical structure of the Antarctic atmosphere. Most studies rely on ground‐based samplers (1.5–15 m height), which may be biased by local boundary layer effects. The recent deployment of UAVs has highlighted significant biological discontinuities between the surface and the free troposphere (Kobayashi 2025), yet characterizing the ‘vertical biogeography’ of the polar atmosphere remains a research priority.

A fourth blind spot concerns the ecological persistence of deposition. While airborne microbes clearly enter soil and snow, few studies trace their long‐term fate. The prevailing evidence suggests that most newcomers are eliminated by the same stringent stressor suite: high UV irradiance, desiccation, freeze–thaw cycling, and extreme oligotrophy that shapes survival during atmospheric transport. Only a small fraction persists, and those survivors are disproportionately enriched for traits conferring oxidative‐stress tolerance and rapid resuscitation capacity, such as the specific genomic adaptations identified in airborne isolates (Dieser et al. 2024). This underscores that deposition acts as a powerful ecological filter: most incoming cells fail, but the few that remain are pre‐adapted to Antarctic selection pressures (Bottos et al. 2014; Wynn‐Williams 1991).

A fundamental limitation across the current Antarctic aerobiological literature is the scale of the findings. Due to extreme logistical challenges, many foundational studies rely on a restricted number of samples. It is crucial to acknowledge that established precepts regarding atmospheric transport may be inadvertently biased by these small sample sizes; consequently, many current concepts could be subject to rapid modification because the overall body of data remains scarce and some evidence is weak. These sample size limitations are further compounded by methodological inconsistencies. Variations in sampling devices and sequencing strategies continue to hamper comparability between studies, a concern raised directly in Antarctic contexts where ultra‐low‐biomass environments are especially vulnerable to contamination and methodological bias (Lopatina et al. 2013). Moving forward, the adoption of standardized approaches and sustained, high‐volume sampling campaigns would enable more integrative, cross‐site analyses and meta‐syntheses (Malard et al. 2022). Furthermore, the taxonomic resolution of these studies, specifically the ones that use metabarcoding, remains bottlenecked by the limitations of current reference databases (Capo et al. 2021). Major genomic repositories suffer from severe cultivation bias, leaving short‐read amplicon sequences frequently lacking closely related database references. Consequently, bioinformatics pipelines often fail to confidently assign taxonomy below the Phylum or Class level (Bokulich et al. 2018). This discrepancy forces assignment algorithms into a compromise: either binning a high proportion of sequences as ‘unclassified’ at lower taxonomic ranks, or erroneously mapping them to distant temperate relatives, thereby generating false positives that obscure true endemic diversity (Edgar 2018; Zinger et al. 2019).

Finally, climate change is predicted to alter transport regimes, storm frequency, and sea‐ice extent, all of which will modulate microbial connectivity (Malard et al. 2022; Zeppenfeld et al. 2021; Moallemi et al. 2021). Predictive frameworks linking circulation shifts, aerosol chemistry, and microbial transport are still absent. Early conceptual work already flagged the role of wind in dispersing biological material (Wynn‐Williams 1991), yet only now are coupled biophysical models becoming feasible. Building such models, for instance, linking long‐term transport cycles (Santibáñez et al. 2018) to biological feedbacks like albedo reduction (Hodson et al. 2017) and aerosol nucleation (Boyer et al. 2025), should therefore be a priority.

### 4.5 Future Directions

Antarctic aerobiology has a coherent framework of three principal pathways, long‐range advection, marine aerosolization, and local resuspension, operating under a chemical–physical scaffold that strongly filters microbial survival (Dall'Osto et al. 2017; Zeppenfeld et al. 2021). Yet several frontiers remain open, and advancing the field will require conceptual integration, methodological innovation, and attention to the broader ecological and climatic context.

A priority is the explicit integration of aerobiology into climate‐change scenarios. Altered circulation patterns are expected to reshape inoculum sources and transport regimes (Moallemi et al. 2021), while shifts in Southern Ocean productivity and sea‐ice retreat will modify the biological signal delivered to Antarctic gateways (Malard et al. 2022). Furthermore, the accumulation of anthropogenic pollutants in the sea‐surface microlayer suggests that future aerobiological inputs will increasingly carry a chemical “fingerprint” of the Anthropocene, potentially selecting for pollutant‐degrading microbial cohorts in coastal aerosols (Martinez‐Varela et al. 2020).

Specific feedback loops require predictive modelling; for instance, changes in penguin colony dynamics could alter regional ammonia emissions, impacting new particle formation and aerosol chemistry (Boyer et al. 2025). Simultaneously, expanding ice‐free ground is predicted to amplify local resuspension, increasing the recycling of terrestrial microbiota across coastal margins (Rosa et al. 2020). Establishing frameworks that link circulation dynamics to microbial transport, modelling how these regional corridors will shift with changing cyclone tracks is essential for predicting the homogenization of Antarctic biodiversity and central to forecasting connectivity under warming scenarios (Galbán et al. 2025).

Methodological innovation is another critical frontier. Amplicon surveys have illuminated community composition but cannot resolve viability or function (Bottos et al. 2014; Lopatina et al. 2013). Emerging genomic approaches now enable strain‐level inference of stress adaptations in ultra‐low‐biomass samples (Dieser et al. 2024), while flow cytometry offers a route to assess cell abundance and integrity in real time (Santibáñez et al. 2018). Furthermore, the adoption of standardized protocols would allow Antarctic datasets to be compared across years and regions, addressing the high heterogeneity currently observed between studies (Malard et al. 2022). Future polar research must also transition toward the generation of ecosystem‐specific reference frameworks. Prioritizing shotgun metagenomics to assemble localized, high‐quality Metagenome‐Assembled Genomes (MAGs) directly from Antarctic environments is essential to anchor unassigned eDNA sequences (Pessi et al. 2023).

Finally, Antarctic aerobiology offers a unique platform to understand planetary protection and astrobiology research. As a uniquely isolated test case for global dispersal, survival here depends primarily on adaptations to oxidative stress, UV radiation, and freeze–thaw cycling (Sterflinger et al. 2012; Junge and Swanson 2008). Insights from these extreme survivors, such as halophilic Archaea capable of persisting in the stratosphere, directly inform models of habitability on other planetary bodies (DasSarma et al. 2017). For example, *Ralstonia* phages identified in Antarctic snow share genetic signatures with strains isolated from spacecraft assembly clean rooms, highlighting their utility as model systems for tracking biological contamination in extraterrestrial analogues. Systematic comparisons between Antarctic aerobiota and those of other extreme environments will help determine the universal limits of life in atmospheric transit (Christner et al. 2008).

## 5 Conclusions

This systematic aerobiological review demonstrates that Antarctica is not an isolated endpoint, but a permeable receptor deeply integrated into the Southern Hemisphere's circulation system. We recognized that aerobiological connectivity is driven by a three‐pathway framework: episodic long‐range advective import, persistent marine aerosolization, and localized resuspension. However, atmospheric transit acts as a severe environmental filter rather than a simple conveyor belt. Extreme UV irradiance, prolonged desiccation, and rapid freeze–thaw cycles strictly select for microorganisms possessing specific resilience traits, such as robust DNA repair mechanisms and the ability to physically shield themselves.

Consequently, successful colonizers represent a low‐flux but functionally profound ecological subsidy. Culture recoveries confirm that a critical fraction of these airborne cells remains metabolically competent upon deposition. By effectively seeding pristine aquatic sinks, these pioneer taxa can establish dominance within local bacterioplankton communities. Furthermore, this atmospheric influx introduces significant multi‐trophic complexity through the co‐dispersal of algae, diverse protists, and viruses, ultimately shaping the local biogeochemical landscape and driving biological interactions over event‐driven and seasonal timescales.

Despite these insights, the field's interpretive power remains constrained by persistent gaps, including pervasive methodological heterogeneity, difficulties in distinguishing viable cells from relic DNA, and a historical neglect of non‐fungal eukaryotes. To transition from observational description to predictive ecosystem forecasting, future research must prioritize coordinated sampling standards and the integration of autonomous technologies, such as UAVs, into polar observing systems. By developing coupled biophysical models and incorporating climate‐change scenarios, the field can definitively quantify how shifting storm tracks and sea‐ice retreat will fundamentally reconfigure the biological source‐sink dynamics shaping Antarctic biodiversity.

## Author Contributions

**Rodrigo Goldenberg‐Barbosa:** conceptualization, methodology, data curation, investigation, validation, writing – review and editing, writing – original draft. **Anna Donato:** writing – review and editing, formal analysis. **Dafne Anjos:** formal analysis, writing – review and editing. **Letícia Eller:** writing – review and editing, formal analysis. **Heitor Evangelista:** writing – review and editing, formal analysis, supervision. **Cesar Amaral:** supervision, writing – review and editing, project administration, writing – original draft.

## Funding

Funding was provided by Coordenação de Aperfeiçoamento de Pessoal de Nível Superior (CAPES); grant number 88887.953802/2024‐0.

## Conflicts of Interest

The authors declare no conflicts of interest.

## Supporting information


**Table S1:** Database search strategies.

**Table S2:** Summary of included studies, detailing sample collection regions, sample types, methodologies, key findings, assigned Antarctic transport pathways, and evidence of organism viability.

**Table S3:** Excluded studies with reasons.

**Data S1:** Supporting Information.

## Data Availability

The data that support the findings of this study are available in the Supporting Information of this article.
