# Supplementary material for: Atmospheric Microbial Transport to Antarctica and Its Ecological Implications: A Systematic Review
Source: Environ Microbiol. 2026 Sep 11;28(9):e70415. doi: 10.1111/1462-2920.70415 (PMC13569471; doi:10.1111/1462-2920.70415)
Supplement: Supplementary file 1 [file EMI-28-e70415-s001.docx]

# Supplementary Table S3. Excluded Studies with Reasons

| Study (Author, Year) | Title / Reference | Reason for Exclusion |
| --- | --- | --- |
| Marina-Montes et al. 2022 | Characterization of atmospheric aerosols in the Antarctic region using Raman Spectroscopy and Scanning Electron Microscopy. | No microbial data |
| Casas et al. 2023 | Inputs, amplification and sinks of perfluoroalkyl substances at coastal Antarctica. | No microbial data |
| Khan et al. 2024 | Soil microbial responses to simulated climate change across polar ecosystems. | No microbial air transport component |
| Guo et al. 2022 | Organic matter distribution in the icy environments of Taylor Valley, Antarctica. | No microbial air transport component |
| Friedmann, 1993 | Extreme environments and exobiology. | No microbial air transport component |
| Teng et al. 2021 | Biogeographic traits of dimethyl sulfide and dimethylsulfoniopropionate cycling in polar oceans. | No microbial air transport component |
| Vick-Majors et al. 2016 | Physiological Ecology of Microorganisms in Subglacial Lake Whillans. | No microbial air transport component |
| Malard et al. 2025 | Daily turnover of airborne bacterial communities in the sub-Antarctic. | No Antarctic focus |
| Rippin et al. 2019 | Cold Acclimation Improves the Desiccation Stress Resilience of Polar Strains of Klebsormidium (Streptophyta). | No microbial air transport component |
| La Ferla et al. 2017 | Prokaryotic Abundance and Activity in Permafrost of the Northern Victoria Land and Upper Victoria Valley (Antarctica). | No microbial air transport component |
| Newsham et al. 2019 | Bacterial Community Composition and Diversity Respond to Nutrient Amendment but Not Warming in a Maritime Antarctic Soil. | No microbial air transport component |
| Gionfriddo et al. 2016 | Microbial mercury methylation in Antarctic sea ice. | No microbial air transport component |
| Pusz et al. 2021 | Airborne fungi in Longyearbyen area (Svalbard, Norway) - case study. | No Antarctic focus |
| Dennis et al. 2019 | Soil bacterial diversity is positively associated with air temperature in the maritime Antarctic. | No microbial air transport component |
| Vestal et al. 1988 | Carbon metabolism of the cryptoendolithic microbiota from the Antarctic desert. | No microbial air transport component |
| King et al. 2024 | Derivation of site-specific environmental quality guideline values for fuel-contaminated soils on sub-Antarctic Macquarie Island. | No microbial data |
| Heinz et al. 2021 | Physicochemical Parameters Limiting Growth of Debaryomyces hansenii in Solutions of Hygroscopic Compounds and Their Effects on the Habitability of Martian Brines. | No microbial air transport component |
| Cerro-Gálvez et al. 2019 | Microbial responses to anthropogenic dissolved organic carbon in the Arctic and Antarctic coastal seawaters. | No microbial air transport component |
| Boenigk et al. 2006 | Evidence for geographic isolation and signs of endemism within a protistan morphospecies. | No microbial air transport component |
| Kawashima et al. 2021 | Ion-exchange resin and denitrification pretreatment for determining δ(15) N-NH(4) (+) , δ(15) N-NO(3) (-) , and δ(18) O-NO(3) (-) values. | No microbial data |
| Mosier et al. 2007 | Microbiota within the perennial ice cover of Lake Vida, Antarctica. | No microbial air transport component |
| Hawrylewicz et al. 1965 | Response of microorganisms to a simulated Martian environment. | No Antarctic focus |
| Siegart et al. 2001 | Physical, chemical and biological processes in Lake Vostok and other Antarctic subglacial lakes | No microbial air transport component |
| Lezcano et al. 2019 | Biomarker Profiling of Microbial Mats in the Geothermal Band of Cerro Caliente, Deception Island (Antarctica): Life at the Edge of Heat and Cold | No microbial air transport component |
| Lauderdale et al. 2024 | Ocean iron cycle feedbacks decouple atmospheric CO(2) from meridional overturning circulation changes. | No microbial data |
| Doran et al. 1994 | Paleolimnology of the McMurdo Dry Valleys, Antarctica. | No microbial air transport component |
| Martinez-Varela et al. 2022 | Polycyclic Aromatic Hydrocarbon Degradation in the Sea-Surface Microlayer at Coastal Antarctica. | No microbial air transport component |
| Sommers et al. 2019 | Comparison of Microbial Communities in the Sediments and Water Columns of Frozen Cryoconite Holes in the McMurdo Dry Valleys, Antarctica. | No microbial air transport component |
| Pakulski et al. 2008 | Effect of stratospheric ozone depletion and enhanced ultraviolet radiation on marine bacteria at Palmer Station, Antarctica in the early austral spring. | No microbial air transport component |
| Priscu et al. 1999 | Geomicrobiology of subglacial ice above Lake Vostok, Antarctica. | No microbial air transport component |
| Karl et al. 1999 | Microorganisms in the accreted ice of Lake Vostok, Antarctica. | No microbial air transport component |
| Delmont et al. 2015 | Genome reconstructions indicate the partitioning of ecological functions inside a phytoplankton bloom in the Amundsen Sea, Antarctica. | No microbial air transport component |
| Wharton et al. 1993 | Stable isotopic biogeochemistry of carbon and nitrogen in a perennially ice-covered Antarctic lake. | No microbial air transport component |
| Newsham et al. 2018 | Discrete taxa of saprotrophic fungi respire different ages of carbon from Antarctic soils. | No microbial air transport component |
| Rohde et al. 2008 | In situ microbial metabolism as a cause of gas anomalies in ice. | No microbial air transport component |
| Bendia et al. 2018 | A Mosaic of Geothermal and Marine Features Shapes Microbial Community Structure on Deception Island Volcano, Antarctica. | No microbial air transport component |
| Zangrando et al. 2020 | Dissolved organic matter in the deep TALDICE ice core: A nano-UPLC-nano-ESI-HRMS method. | No microbial air transport component |
| Hirsch, 1986 | Microbial life at extremely low nutrient levels. | No microbial air transport component |
| Lutz et al. 2019 | The Biodiversity and Geochemistry of Cryoconite Holes in Queen Maud Land, East Antarctica. | No microbial air transport component |
| Siegert, 2000 | The identification, examination and exploration of Antarctic subglacial lakes. | No microbial air transport component |
| Snape et al. 2005 | Investigation of evaporation and biodegradation of fuel spills in Antarctica. I. A chemical approach using GC-FID. | No microbial air transport component |
| Friedmann and Kibler, 1980 | Nitrogen economy of endolithic microbial communities in hot and cold deserts. | No microbial air transport component |
| Boniek et al. 2010 | Characterization of bacterial strains capable of desulphurisation in soil and sediment samples from Antarctica. | No microbial air transport component |
| Ghimire et al. 2020 | Comparative Genomic Study of Polar Lichen-Associated Hymenobacter sp. PAMC 26554 and Hymenobacter sp. PAMC 26628 Reveals the Presence of Polysaccharide-Degrading Ability Based on Habitat. | No microbial air transport component |
| Kochkina et al. 2001 | Micromycetes and actinobacteria under conditions of many years of natural cryopreservation | No microbial air transport component |
| Rappazzo et al. 2025 | PCB-oxidizing bacteria in Arctic and Antarctic lakes: insights into bioremediation potential in polar ecosystems | No microbial air transport component |
| Shaffer et al. 2025 | Multi-technique characterization of iron reduction by an Antarctic Shewanella: an analog system for putative Martian biosignature identification | No microbial air transport component |
| Retallack, 2025 | A short history of astropedology | No Antarctic focus |
| Vaqué et al. 2025 | Assessing the relationship between viruses and protists and their role in dimethylsulphoniopropionate release in Antarctic surface microlayers | No microbial air transport component |
| Han et al. 2024 | Unveiling unique microbial nitrogen cycling and nitrification driver in coastal Antarctica | No microbial air transport component |
| Bhattacharjee et al. 2024 | Do microplastics accumulate in penguin internal organs? Evidence from Svenner island, Antarctica | No microbial air transport component |
| McKay et al. 2024 | Pore size distribution and water retention in colonized Antarctic Beacon sandstone | No microbial air transport component |
| Jiang et al. 2024 | Molecular Composition Evolution of Dissolved Organic Matter With Water Depth in Prydz Bay of East Antarctic: Carbon Export Implications | No microbial air transport component |
| Bargagli and Rota, 2024 | Environmental contamination and climate change in Antarctic ecosystems: an updated overview | No microbial air transport component |
| Acuña-Rodríguez et al. 2024 | The role of the soil microbiome in the colonisation of glacier forefields by Antarctic pearlwort (Colobanthus quitensis) under current and future climate change scenario | No microbial air transport component |
| Latorre et al. 2023 | Plankton metabolic balance in the eastern Beagle Channel during spring | No Antarctic focus |
| Cimino et al. 2023 | Long-term patterns in ecosystem phenology near Palmer Station, Antarctica, from the perspective of the Adélie penguin | No microbial air transport component |
| Corkill et al. 2023 | Physical and Biogeochemical Properties of Rotten East Antarctic Summer Sea Ice | No microbial data |
| Swadling et al. 2023 | Biological responses to change in Antarctic sea ice habitats | No microbial air transport component |
| Wang et al. 2023 | The phylogeny and metabolic potentials of an n-alkane-degrading Venatorbacter bacterium isolated from deep-sea sediment of the Mariana Trench | No microbial air transport component |
| Tibbett et al. 2022 | Cenozoic Antarctic Peninsula Temperatures and Glacial Erosion Signals From a Multi-Proxy Biomarker Study | No microbial data |
| Fan et al. 2022 | Aerosol iron speciation and seasonal variation of iron oxidation state over the western Antarctic Peninsula | No microbial data |
| Schmidt et al. 2022 | Microbial biogeochemistry and phosphorus limitation in cryoconite holes on glaciers across the Taylor Valley, McMurdo Dry Valleys, Antarctica | No microbial air transport component |
| Wang et al. 2022 | Research progress of cryoconite microorganisms and biogeochemical cycling of carbon and nitrogen driven by cryoconite in tripolar glaciers | Foreign language |
| Lu et al. 2021 | Paleoenvironmental Significance of Magnetofossils in Pelagic Sediments in the Equatorial Pacific Ocean Before and After the Eocene/Oligocene Boundary | No microbial data |
| Liu et al. 2021 | Bacteria in the lakes of the Tibetan Plateau and polar regions | No microbial air transport component |
| George et al. 2021 | Antarctic Water Tracks: Microbial Community Responses to Variation in Soil Moisture, pH, and Salinity | No microbial air transport component |
| Polyakov et al. 2021 | Evaluation of stabilization rate of high and low molecular organic matter in cryoconite holes from the Arctic, Antarctic and Caucasus mountain ecosystems by 13C-NMR spectroscopy | No microbial data |
| Chou et al. 2021 | Organic sulfones in the brine of Lake Vida, East Antarctica | No microbial data |
| Kim et al. 2020 | Soil water content as a critical factor for stable bacterial community structure and degradative activity in maritime Antarctic soil | No microbial air transport component |
| Zhang et al. 2020 | Methyl Chloride and Methyl Bromide Production and Consumption in Coastal Antarctic Tundra Soils Subject to Sea Animal Activities | No microbial data |
| Wang et al. 2020 | Identification of a functional dddD-Rh for dimethyl sulfide production in the Antarctic Rhodococcus sp. NJ-530 | No microbial air transport component |
| Rodríguez-Ros et al. 2020 | Distribution and drivers of marine isoprene concentration across the Southern Ocean | No microbial data |
| Horrocks et al. 2020 | Predicting climate change impacts on maritime Antarctic soils: a space-for-time substitution study | No microbial air transport component. |
| Li et al. 2020 | Methane production in the oxygenated water column of a perennially ice-covered Antarctic lake | No microbial air transport component. |
| Azzoni et al. 2018 | Bacterial diversity in snow from mid-latitude mountain areas: Alps, Eastern Anatolia, Karakoram and Himalaya | No Antarctic focus |
| Ahmad et al. 2018 | Effect of heavy metals on biodegradation of phenol by antarctic bacterium: Arthrobacter bambusae strain AQ5-003 | No microbial air transport component. |
| Collins et al. 2018 | The molecular products and biogeochemical significance of lipid photooxidation in West Antarctic surface waters | No microbial air transport component |
| Redeker et al. 2017 | Microbial metabolism directly affects trace gases in (sub) polar snowpacks | No microbial air transport component |
| Michaud et al. 2017 | Microbial oxidation as a methane sink beneath the West Antarctic Ice Sheet | No microbial air transport component |
| Asher et al. 2017 | Processes driving seasonal variability in DMS, DMSP, and DMSO concentrations and turnover in coastal Antarctic waters | No microbial air transport component |
| Retallack et al. 2016 | Archean coastal-plain paleosols and life on land | No Antarctic focus |
| Davies et al. 2016 | Diploptene δ13C values from contemporary thermokarst lake sediments show complex spatial variation | No Antarctic focus |
| Parrilli et al. 2016 | Large-scale biofilm cultivation of Antarctic bacterium Pseudoalteromonas haloplanktis TAC125 for physiologic studies and drug discovery | No microbial air transport component |
| Newsham et al. 2016 | Relationship between soil fungal diversity and temperature in the maritime Antarctic | No microbial air transport component |
| Goordial et al. 2016 | Cold adaptive traits revealed by comparative genomic analysis of the eurypsychrophile Rhodococcus sp. JG3 isolated from high elevation McMurdo Dry Valley permafrost, Antarctica | No microbial air transport component |
| Onofri et al. 2015 | Survival of Antarctic Cryptoendolithic Fungi in Simulated Martian Conditions on Board the International Space Station | No microbial air transport component |
| Gilichinsky et al. 2015 | Morphometry of volcanic cones on Mars in perspective of Astrobiological Research | No Antarctic focus |
| Cornejo-D’Ottone et al. 2015 | An unaccounted for N2O sink in the surface water of the eastern subtropical South Pacific: Physical versus biological mechanisms | No microbial air transport component |
| Camacho et al. 2015 | Total mercury and methyl-mercury contents and accumulation in polar microbial mats | No microbial air transport component |
| Zhan et al. 2015 | Novel N4 bacteriophages prevail in the cold biosphere | No microbial air transport component |
| Saba et al. 2014 | Winter and spring controls on the summer food web of the coastal West Antarctic Peninsula | No microbial data |
| Telling et al. 2014 | Spring thaw ionic pulses boost nutrient availability and microbial growth in entombed Antarctic Dry Valley cryoconite holes | No microbial air transport component |
| Wisnieski et al. 2014 | Characterization of sources and temporal variation in the organic matter input indicated by n-alkanols and sterols in sediment cores from Admiralty Bay, King George Island, Antarctica | No microbial air transport component |
| Pancost et al. 2013 | Early Paleogene evolution of terrestrial climate in the SW Pacific, Southern New Zealand | No Antarctic focus |
| Stanish et al. 2013 | Environmental factors influencing diatom communities in Antarctic cryoconite holes | No microbial air transport component |
| Braslavsky et al. 2013 | LabVIEW-operated novel nanoliter osmometer for ice binding protein investigations. | No microbial data |
| Björck et al. 2012 | Multi-proxy analyses of a peat bog on Isla de los Estados, easternmost Tierra del Fuego: A unique record of the variable Southern Hemisphere Westerlies since the last deglaciation | No Antarctic focus |
| Izaguirre et al. 2012 | Responses of a Maritime Antarctic lake to a catastrophic draining event under a climate change scenario | No microbial air transport component |
| Tamppari et al. 2012 | Effects of extreme cold and aridity on soils and habitability: McMurdo Dry Valleys as an analogue for the Mars Phoenix landing site | No microbial air transport component |
| Lantuit et al. 2012 | Permafrost and man | No Antarctic focus |
| de Siqueira Campos et al. 2011 | Antarctic - South American interactions in the Marine environment: A COMARGE and CAML effort through the South American Consortium on Antarctic Marine biodiversity | No microbial data |
| Timmery et al. 2011 | Characterization of Bacilli isolated from the confined environments of the Antarctic Concordia station and the International Space Station | No microbial air transport component |
| Bárcena et al. 2011 | Spatial patterns of soil development, methane oxidation, and methanotrophic diversity along a receding Glacier forefield, Southeast Greenland | No Antarctic focus |
| Bomfleur et al. 2011 | Macrofossil evidence for pleuromeialean lycophytes from the triassic of Antarctica | No microbial data |
| Chuvochina et al. 2011 | Three events of Saharan dust deposition on the Mont Blanc glacier associated with different snow-colonizing bacterial phylotypes | No Antarctic focus |
| Han et al. 2011 | Optimization of cold-active chitinase production from the Antarctic bacterium, Sanguibacter antarcticus KOPRI 21702 | No microbial air transport component |
| Peterse et al. 2011 | Decoupled warming and monsoon precipitation in East Asia over the last deglaciation | No Antarctic focus |
| Peterse et al. 2010 | Biogeography of terrestrial cyanobacteria from Antarctic ice-free areas | No microbial air transport component |
| Priscu et al. 2010 | Probing subglacial environments under the Whillans ice stream | No microbial air transport component |
| Wang et al. 2010 | Late autumn to spring changes in the inorganic and organic carbon dissolved in the water column at Scholaert Channel, West Antarctica | No microbial data |
| Hodgson et al. 2009 | Exploring former subglacial Hodgson Lake, Antarctica Paper I: site description, geomorphology and limnology | No microbial air transport component |
| Mahaney et al. 2009 | Secondary Fe and Al in Antarctic paleosols: Correlation to Mars with prospect for the presence of life | No microbial data |
| Fritsen et al. 2008 | Inter-annual sea-ice dynamics and micro-algal biomass in winter pack ice of Marguerite Bay, Antarctica | No microbial air transport component |
| Walker et al. 2008 | Long-term experimental warming alters nitrogen-cycling communities but site factors remain the primary drivers of community structure in high arctic tundra soils | No Antarctic focus |
| Schouten et al. 2008 | Onset of long-term cooling of Greenland near the Eocene-Oligocene boundary as revealed by branched tetraether lipids | No Antarctic focus |
| Bagshaw et al. 2007 | Biogeochemical evolution of cryoconite holes on Canada Glacier, Taylor Valley, Antarctica | No microbial air transport component |
| Foreman et al. 2007 | Metabolic activity and diversity of cryoconites in the Taylor Valley, Antarctica | No microbial air transport component |
| Bargagli et al. 2007 | Environmental biogeochemistry of mercury in Antarctic ecosystems | No microbial air transport component |
| Vlasov et al. 2006 | Micromycetes from the Polar Stations area in western Antarctica | Foreign Language |
| Aislabie et al. 2006 | Dominant bacteria in soils of Marble Point and Wright Valley, Victoria Land, Antarctica | No microbial air transport component |
| Barrett et al. 2006 | Terrestrial ecosystem processes of Victoria Land, Antarctica | No microbial air transport component |
| Bárcena et al. 2006 | Holocene neoglacial events in the Bransfield Strait (Antarctica). Palaeocenographic and palaeoclimatic significance | No microbial data |
| Christner et al. 2006 | Limnological conditions in subglacial Lake Vostok, Antarctica | No microbial air transport component |
| Abyzov et al. 2006 | Super-long anabiosis of ancient microorganisms in ice and terrestrial models for development of methods to search for life on Mars, Europa and other planetary bodies | No Antarctic focus |
| Panikov et al. 2006 | Microbial activity in soils frozen to below -39°c | No Antarctic focus |
| Sorokhtin and Semenova, 2005 | Role of nitrogen-fixing bacteria in the origin of the Earth glaciation | No Antarctic focus |
| Hodgson et al. 2005 | Late Pleistocene record of elevated UV radiation in an Antarctic lake | No microbial air transport component |
| Zdanowski et al. 2005 | Bacterial role in the decomposition of marine-derived material (penguin guano) in the terrestrial maritime Antarctic | No microbial air transport component |
| Dickhut et al. 2005 | Effects of temperature warming during a bioremediation study of natural and nutrient-amended hydrocarbon-contaminated sub-Antarctic soils | No microbial data |
| Delille et al. 2004 | Effects of temperature warming during a bioremediation study of natural and nutrient-amended hydrocarbon-contaminated sub-Antarctic soils | No Antarctic focus |
| Tranter et al. 2004 | Extreme hydrochemical conditions in natural microcosms entombed within Antarctic ice | No microbial air transport component |
| Moran et al. 2003 | Linking a bacterial taxon to sulfur cycling in the sea: Studies of the marine Roseobacter group | No microbial air transport component |
| Fiala et al. 2003 | Mesoscale surface distribution of biogeochemical characteristics in the Crozet Basin frontal zones (South Indian Ocean) | No Antarctic focus |
| Hobbie, 2003 | Scientific accomplishments of the Long Term Ecological Research program: An introduction | No Antarctic focus |
| Price et al. 2002 | Temperature profile for glacial ice at the South Pole: Implications for life in a nearby subglacial lake | No microbial air transport component |
| Anderson et al. 2002 | The Southern Ocean's biological pump during the Last Glacial Maximum | No microbial data |
| Jahic et al. 2002 | Modeling of growth and energy metabolism of Pichia pastoris producing a fusion protein | No Antarctic focus |
| Sowers, 2001 | N2O record spanning the penultimate deglaciation from the Vostok ice core | No microbial air transport component |
| Boucot et al. 2001 | A critique of phanerozoic climatic models involving changes in the CO2 content of the atmosphere | No Antarctic focus |
| Kochkina et al. 2001 | Survival of micromycetes and actinobacteria under conditions of long-term natural cryopreservation | No microbial air transport component |
| Cockell et al. 2001 | The history of the UV radiation climate of the earth-Theoretical and space-based observations | No Antarctic focus |
| McKay et al. 2001 | The physics, biology, and environmental ethics of making mars habitable | No Antarctic focus |
| Bréas et al. 2001 | The global methane cycle: Isotopes and mixing ratios, sources and sinks | No Antarctic focus |
| Mahaney et al. 2001 | Morphogenesis of antarctic paleosols: Martian analogue |  |
| Carrillo et al. 1999 | Dissolved inorganic carbon pool dynamics in northern Gerlache Strait, Antarctica | No microbial air transport component |
| Pongratz et al. 1999 | Production of methylated mercury, lead, and cadmium by marine bacteria as a significant natural source for atmospheric heavy metals in polar regions | No microbial air transport component |
| Hawes et al. 1999 | Environmental conditions during freezing, and response of microbial mats in ponds of the McMurdo Ice Shelf, Antarctica | No microbial air transport component |
| Roten et al. 1998 | Impact resistance of bacteria entrapped in small meteorites | No Antarctic focus |
| Ingole et al. 1998 | Ecobiological assessment of a freshwater lake at Schirmacher Oasis, East Antarctica, with reference to human activities | No microbial air transport component |
| Helbling et al. 1992 | Impact of natural ultraviolet radiation on rates of photosynthesis and on specific marine phytoplankton species | No microbial air transport component |
| Baker et al. 1991 | Microbial degradation of methanesulphonic acid: A missing link in the biogeochemical sulphur cycle | No microbial data |
| Maki et al. 1990 | Biofilm formation on metal surfaces in antarctic waters | No microbial air transport component |
| Bonani et al. 1988 | Preliminary report on radiocarbon dating of cryptoendolithic microorganisms. | No microbial air transport component |
| François et al. 1988 | Ozone, climate and biospheric environment in the ancient oxygen-poor atmosphere | No Antarctic focus |
| McKay et al. 1984 | Continuous temperature measurements in the cryptoendolithic microbial habitat by satellite-relay data acquisition system ( Antarctica). | No microbial air transport component |
| Vincent et al. 1981 | Nitrous oxide cycling in Lake Vanda, Antarctica | No microbial data |
| Levin et al. 1981 | A search for a nonbiological explanation of the Viking Labeled Release life detection experiment | No microbial air transport component |
| Inoue, 1976 | Quantitative ecology of microorganisms of syowa station in antarctica and isolation of psychrophiles | No microbial air transport component |
| Meyer et al. 1962 | Viable micro-organisms in a fifty-year-old yeast preparation in Antarctica | No microbial air transport component |
| Hughes et al. 2013 | Microbial control of bromocarbon concentrations in coastal waters of the western Antarctic Peninsula | No microbial air transport component |
| de Angelis et al. 2013 | Micro-investigation of EPICA Dome C bottom ice: Evidence of long term in situ processes involving acid-salt interactions, mineral dust, and organic matter | No microbial air transport component |
| Kaur et al. 2013 | Role of coreless winter in favoring survival of microorganisms over the schirmacher Oasis, East Antarctica | No microbial air transport component |
| Jackson et al. 2015 | Global patterns and environmental controls of perchlorate and nitrate co-occurrence in arid and semi-arid environments | No Antarctic focus |
| Biondi et al. 2008 | Cyanobacteria from benthic mats of Antarctic lakes as a source of new bioactivities | No microbial air transport component |
| Havig et al. 2019 | Hypolithic photosynthesis in hydrothermal areas and implications for cryptic oxygen oases on archean continental surfaces | No Antarctic focus |
| Zepp et al. 2003 | Interactive effects of ozone depletion and climate change on biogeochemical cycles | No Antarctic focus |
| Aslanov et al. 2024 | Pathogenic potential of ornithogenic Escherichia coli strains detected in the Earth's polar regions | No microbial air transport component |
| Núñez et al. 2006 | Modelled effects of ambient UV radiation on a natural Antarctic marine microbial community | No microbial air transport component |
| Celussi et al., 2009 | Short-term under-ice variability of prokaryotic plankton communities in coastal Antarctic waters (Cape Hallett, Ross Sea) | No microbial air transport component |
| Noh et al., 2020 | Microbiome in Cladonia squamosa Is Vertically Stratified According to Microclimatic Conditions | No microbial air transport component |
| Leotta et al., 2003 | Avian cholera in a southern giant petrel (Macronectes giganteus) from Antarctica | No microbial air transport component |
| Studinger et al., 2002 | Ice cover, landscape setting, and geological framework of Lake Vostok, East Antarctica | No microbial air transport component |
| McKay et al., 1985 | The cryptoendolithic microbial environment in the Antarctic cold desert: temperature variations in nature. | No microbial air transport component |
| Pearce et al., 2016 | Aerobiology Over Antarctica - A New Initiative for Atmospheric Ecology. | No microbial data |
| Ohno et al., 2016 | Physicochemical properties of bottom ice from Dome Fuji, inland East Antarctica | No microbial air transport component |
| Ji et al., 2017 | Atmospheric trace gases support primary production in Antarctic desert surface soil | No microbial air transport component |
| Sannino et al., 2016 | Atmospheric trace gases support primary production in Antarctic desert surface soil | No microbial air transport component |
| Gesheva et al., 2010 | Biosurfactant production by halotolerant rhodococcus fascians from Casey Station, Wilkes Land, antarctica | No microbial air transport component |
| Boyer et al., 2025 | Penguin guano is an important source of climate-relevant aerosol particles in Antarctica | No microbial data |
| Echeveste et al., 2024 | Airborne organic pollutants impact microbial communities in temperate and Antarctic seawaters. | No microbial air transport component |
| Convey et al., 2018 | The importance of understanding annual and shorter-term temperature patterns and variation in the surface levels of polar soils for terrestrial biota | No microbial data |
| Sterflinger et al., 2012 | Fungi in hot and cold deserts with particular reference to microcolonial fungi | No microbial air transport component |
| Zeppenfeld et al., 2021 | Sea Ice Microbiota in the Antarctic Peninsula Modulates Cloud-Relevant Sea Spray Aerosol Production | No microbial air transport component |
| Decesari et al., 2020 | Shipborne measurements of Antarctic submicron organic aerosols: An NMR perspective linking multiple sources and bioregions | No microbial data |
| Zhou et al., 2016 | The impact of dissolved organic carbon and bacterial respiration on pCO2 in experimental sea ice | No microbial air transport component |
| Van Houdt et al., 2008 | Evaluation of the airborne bacterial population in the periodically confined Antarctic base Concordia. | No microbial air transport component |
| Pérez et al., 2022 | Diazotrophic activity and denitrification in two long-term chronosequences of maritime Antarctica | No microbial air transport component |
| Dall’Osto et al., 2017 | Antarctic sea ice region as a source of biogenic organic nitrogen in aerosols. | No microbial air transport component |
| Dall’Osto et al., 2019 | Simultaneous Detection of Alkylamines in the Surface Ocean and Atmosphere of the Antarctic Sympagic Environment | No microbial air transport component |
| Dall’Osto et al., 2026 | Quality over Quantity: Organic Compounds Altering the Antarctic  Sea Spray Aerosol Concentrations | No microbial air transport component |
| Sun et al., 2026 | Putative drivers of maritime Antarctic soil resistomes in the early 21st century: A baseline for monitoring environmental change and human influence | No microbial air transport component |

This table lists all studies that were excluded from the final synthesis, following the first screening titles/abstracts and a second screening of full-texts, with concise reasons for exclusion. This fulfills PRISMA 2020 Item #16b requirements for transparent reporting of study selection. Note: Duplicates are accounted for in the PRISMA flow diagram but not individually listed.
