# Supplementary material for: Atmospheric Microbial Transport to Antarctica and Its Ecological Implications: A Systematic Review
Source: Environ Microbiol. 2026 Sep 11;28(9):e70415. doi: 10.1111/1462-2920.70415 (PMC13569471; doi:10.1111/1462-2920.70415)
Supplement: Supplementary file 2 [file EMI-28-e70415-s002.docx]

# PRISMA 2020 Checklist — Antarctic Atmospheric Microbiota Systematic Review

Based on Page et al. (BMJ, 2021). This checklist summarizes compliance with PRISMA 2020 items for the manuscript submitted to Environmental Microbiology.

| Section | Item # and Name | PRISMA 2020 Description | How This Review Meets the Requirement |
| --- | --- | --- | --- |
| TITLE | 1. Title | Identify the report as a systematic review. | Title includes the term 'systematic review'. |
| ABSTRACT | 2. Abstract | See the PRISMA 2020 for Abstracts checklist. | Abstract provided outlining the background, methods, results (three-pathway framework), and conclusions. |
| INTRODUCTION | 3. Rationale | Describe the rationale for the review in the context of what is already known. | Rationale described: the field lacks a comprehensive synthesis of current knowledge and identification of key research gaps. |
| INTRODUCTION | 4. Objectives | Provide an explicit statement of the objective(s) or question(s) the review addresses. | Objectives defined as: (1) transport mechanisms, (2) survival/adaptation, (3) ecological impacts, and (4) knowledge gaps. |
| METHODS | 5. Eligibility criteria | Specify inclusion and exclusion criteria for the review and how studies were grouped for syntheses. | Detailed in Methods (English studies, 1991-2026, microbial focus); exclusion reasons summarized in the PRISMA flow diagram (Figure 1). |
| METHODS | 6. Information sources | Specify all databases, registers, websites, organizations, reference lists, and other sources searched or consulted to identify studies. Specify the date when each source was last searched or consulted. | Scopus and PubMed searched for literature spanning 1991 to 2026. (Table S1). |
| METHODS | 7. Search strategy | Present the full search strategies for all databases, including filters and limits, used so that they could be repeated. | Combined search terms for both databases are detailed in Table S1. |
| METHODS | 8. Selection process | Specify the methods used to decide whether a study met the inclusion criteria of the review, including how many reviewers screened each record and each report retrieved, whether they worked independently, and if applicable, details of automation tools used. | Titles and abstracts were screened for relevance, followed by full-text assessment (Figure 1). |
| METHODS | 9. Data collection process | Specify the methods used to collect data from reports, including how many reviewers collected data, whether they worked independently, and any processes for obtaining or confirming data from study investigators. | Data extraction details are reported and summarized in Table 1 and Tables S1–S3. |
| METHODS | 10. Data items | List and define all outcomes for which data were sought. | Variables extracted: geographic distribution, methodologies applied, transport pathways, viability assessment, temporal distribution, and sample types (Figure 2) |
| METHODS | 11. Study risk of bias assessment | Specify the methods used to assess risk of bias in the included studies, including details of the tool(s) used. | Not formally assessed with a scoring tool; methodological biases (e.g., contamination, viability markers) are discussed qualitatively. |
| METHODS | 12. Effect measures | Specify for each outcome the effect measure(s) (e.g., risk ratio, mean difference) used in the synthesis or presentation of results. | Not applicable (qualitative synthesis only). |
| METHODS | 13. Synthesis methods | Describe the processes used to decide which studies were eligible for each synthesis, methods of data synthesis, and any sensitivity analyses. | Studies were analyzed and grouped into four qualitative themes: transport mechanisms, survival and adaptation, ecological impacts, and research gaps. |
| METHODS | 14. Reporting bias assessment | Describe any methods used to assess risk of bias due to missing results in a synthesis. | Not formally assessed; potential publication and cultivation biases are discussed in the limitations/gaps section. |
| METHODS | 15. Certainty assessment | Describe any methods used to assess certainty (or confidence) in the body of evidence for an outcome. | Discussed qualitatively; evidence weighted by methodological robustness (e.g., distinguishing viable cells from relic DNA). |
| RESULTS | 16. Study selection | Describe the results of the search and selection process, ideally using a flow diagram. | PRISMA flow diagram (Figure 1) and narrative summary provided. |
| RESULTS | 17. Study characteristics | Cite each included study and present its characteristics. | Representative studies are in Table 1; the complete dataset of the 53 included studies is provided in Table S2. |
| RESULTS | 18. Risk of bias in studies | Present assessments of risk of bias for each included study. | Discussed qualitatively in the context of methodological inconsistencies and ultra-low-biomass contamination risks. |
| RESULTS | 19. Results of individual studies | For all outcomes, present, for each study, summary data and effect estimates. | Key findings summarized in Table 1 and narrative text. |
| RESULTS | 20. Results of syntheses | Summarize the main findings, including evidence strength, limitations, and gaps. | Summarized by transport mechanism (advection, aerosolization, resuspension) and viability evidence. |
| DISCUSSION | 21. Summary of evidence | Summarize main findings including the strength of evidence for each main outcome. | Discussion synthesizes evidence on transport sources, survival/stressors, and ecological deposition processes. |
| DISCUSSION | 22. Limitations of evidence | Discuss limitations of the evidence included in the review. | Noted low viability testing (52.8% lacked direct evidence) and sparse spatial coverage in the continental interior. |
| DISCUSSION | 23. Limitations of review processes | Discuss limitations of the review processes used. | Acknowledged inclusion of only English-language studies. |
| DISCUSSION | 24. Conclusions | Provide a general interpretation of the results in the context of other evidence. | Conclusions link microbial atmospheric transit to severe environmental filtering and climate-driven source-sink dynamics. |
| OTHER INFORMATION | 25. Registration and protocol | Provide registration information for the review. | Not preregistered; review process declared in Methods. |
| OTHER INFORMATION | 26. Support | Describe sources of financial or non-financial support. | Institutional and personal funding (CAPES) acknowledged. |
| OTHER INFORMATION | 27. Availability of data, code, and materials | Indicate where data, code, and other materials are available. | All extracted data are available within the study or in the Supporting Information files. |
