# Supplementary material for: Atmospheric Microbial Transport to Antarctica and Its Ecological Implications: A Systematic Review
Source: Environ Microbiol. 2026 Sep 11;28(9):e70415. doi: 10.1111/1462-2920.70415 (PMC13569471; doi:10.1111/1462-2920.70415)
Supplement: Supplementary file 3 [file EMI-28-e70415-s003.docx]

# Supplementary Table S1. Database Search Strategies

This table presents the finalized search strategies used in each database for the systematic review of atmospheric microbial transport to Antarctica. Searches were conducted in February 2026, limited to peer-reviewed journal articles in English. No automation tools were used for screening or deduplication.

| Database | Search String / Query | Filters and Notes |
| --- | --- | --- |
| Scopus | TITLE-ABS-KEY ( ( antarctica OR antarctic ) AND ( "airborne" OR "aerial transport" OR "atmospheric transport" OR "long-range transport" OR "atmospheric deposition" OR aerosol* OR "air" OR "atmosphere" ) AND ( microorganism* OR microbe* OR bacteria OR microbiota OR microbiome ) ) AND ( LIMIT-TO ( DOCTYPE , "ar" ) ) | Search fields: TITLE-ABS-KEY. Document type: Article and Review. Language: English. Date range: Database inception – September 2025. Exported in CSV format for manual screening and deduplication. |
| PubMed | ((antarctica[Title/Abstract] OR antarctic[Title/Abstract]) AND ("airborne"[Title/Abstract] OR "aerial transport"[Title/Abstract] OR "atmospheric transport"[Title/Abstract] OR "long-range transport"[Title/Abstract] OR "atmospheric deposition"[Title/Abstract] OR aerosol*[Title/Abstract] OR "air"[Title/Abstract] OR "atmosphere"[Title/Abstract]) AND (microorganism*[Title/Abstract] OR microbe*[Title/Abstract] OR bacteria[Title/Abstract] OR microbiota[Title/Abstract] OR microbiome[Title/Abstract])) | Filters: Journal Articles only. Language: English. Search conducted: September 10, 2025. No species or subject restrictions applied. Results exported and screened manually according to inclusion/exclusion criteria (Table S4). |

The same Boolean structure was applied consistently across databases to ensure comprehensive coverage of literature on microbial transport and atmospheric deposition processes in Antarctic contexts.
